# Subclinical atherosclerosis and its progression are modulated by *PLIN2* through a feed‐forward loop between LXR and autophagy

**DOI:** 10.1111/joim.12951

**Published:** 2019-07-29

**Authors:** P. Saliba‐Gustafsson, M. Pedrelli, K. Gertow, O. Werngren, V. Janas, S. Pourteymour, D. Baldassarre, E. Tremoli, F. Veglia, R. Rauramaa, A.J. Smit, P. Giral, S. Kurl, M. Pirro, U. de Faire, S.E. Humphries, A. Hamsten, the IMPROVE Study Group, C. R. Sirtori, S. Castelnuovo, M. Amato, B. Frigerio, A. Ravani, D. Sansaro, C. Tedesco, F. Bovis, A. Discacciati, M. Ahl, G. Blomgren, M. J. Eriksson, P. Fahlstadius, M. Heinonen, L. Nilson, J. Cooper, J. Acharya, K. Huttunen, E. Rauramaa, H. Pekkarinen, I. M. Penttila, J. Torronen, A. I. van Gessel, A. M. van Roon, G. C. Teune, W. D. Kuipers, M. Bruin, A. Nicolai, P. Haarsma‐Jorritsma, D. J. Mulder, H. J. G. Bilo, G. H. Smeets, J. L. Beaudeux, J. F. Kahn, V. Carreau, A. Kontush, J. Karppi, T. Nurmi, K. Nyyssonen, R. Salonen, T. P. Tuomainen, J. Tuomainen, J. Kauhanen, G. Vaudo, A. Alaeddin, D. Siepi, G. Lupattelli, G. Schillaci, I. Gonçalves, M. Orho‐Melander, A. Franco‐Cereceda, J. Borén, P. Eriksson, J. Magné, P. Parini, E. Ehrenborg

**Affiliations:** ^1^ Cardiovascular Medicine Unit, Department of Medicine, Center for Molecular Medicine at BioClinicum Karolinska University Hospital, Karolinska Institutet Stockholm Sweden; ^2^ Cardiovascular Medicine Stanford University School of Medicine Palo Alto California USA; ^3^ Division of Clinical Chemistry, Department of Laboratory Medicine Karolinska Institutet Huddinge Huddinge Sweden; ^4^ Department of Medical Biotechnology and Translational Medicine Università degli Studi di Milano Milan Italy; ^5^ Centro Cardiologico Monzino, IRCCS Milan Italy; ^6^ Dipartimento di Scienze Farmacologiche e Biomolecolari Università di Milano Milan Italy; ^7^ Foundation for Research in Health Exercise and Nutrition Kuopio Research Institute of Exercise Medicine Kuopio Finland; ^8^ Department of Medicine University Medical Center Groningen Groningen The Netherlands; ^9^ Assistance Publique Hopitaux de Paris, Service Endocrinologie‐Metabolisme, Groupe Hospitalier Pitie‐Salpetriere Unites de Prevention Cardiovasculaire Paris France; ^10^ Institute of Public Health and Clinical Nutrition University of Eastern Finland Kuopio Finland; ^11^ Unit of Internal Medicine, Angiology and Arteriosclerosis Diseases, Department of Medicine University of Perugia Perugia Italy; ^12^ Division of Cardiovascular Epidemiology, Institute of Environmental Medicine Karolinska Institutet Stockholm Sweden; ^13^ Centre for Cardiovascular Genetics, Institute Cardiovascular Science University College London London UK; ^14^ Experimental Cardiovascular Research Group and Cardiology Department, Clinical Research Center, Clinical Sciences Malmö Lund University Lund Sweden; ^15^ Department of Clinical Sciences in Malmö, Lund University Diabetes Centre Lund University Lund Sweden; ^16^ Cardiothoracic Surgery Unit, Department of Molecular Medicine and Surgery Karolinska Institutet at Karolinska University Hospital Solna Solna Sweden; ^17^ Department of Molecular and Clinical Medicine/Wallenberg Laboratory University of Gothenburg and Sahlgrenska University Hospital Gothenburg Sweden; ^18^ St Jude Children’s Research Hospital Department of Immunology Memphis Tennessee USA; ^19^ Metabolism Unit, Department of Medicine Karolinska Institutet at Karolinska University Hospital Huddinge Huddinge Sweden

**Keywords:** 27OH‐cholesterol, atherosclerosis, autophagy, liver‐X‐receptor, PLIN2

## Abstract

**Background:**

Hyperlipidaemia is a major risk factor for cardiovascular disease, and atherosclerosis is the underlying cause of both myocardial infarction and stroke. We have previously shown that the Pro251 variant of perilipin‐2 reduces plasma triglycerides and may therefore be beneficial to reduce atherosclerosis development.

**Objective:**

We sought to delineate putative beneficial effects of the Pro251 variant of perlipin‐2 on subclinical atherosclerosis and the mechanism by which it acts.

**Methods:**

A pan‐European cohort of high‐risk individuals where carotid intima‐media thickness has been assessed was adopted. Human primary monocyte‐derived macrophages were prepared from whole blood from individuals recruited by perilipin‐2 genotype or from buffy coats from the Karolinska University hospital blood central.

**Results:**

The Pro251 variant of perilipin‐2 is associated with decreased intima‐media thickness at baseline and over 30 months of follow‐up. Using human primary monocyte‐derived macrophages from carriers of the beneficial Pro251 variant, we show that this variant increases autophagy activity, cholesterol efflux and a controlled inflammatory response. Through extensive mechanistic studies, we demonstrate that increase in autophagy activity is accompanied with an increase in liver‐X‐receptor (LXR) activity and that LXR and autophagy reciprocally activate each other in a feed‐forward loop, regulated by *CYP27A1* and 27OH‐cholesterol.

**Conclusions:**

For the first time, we show that perilipin‐2 affects susceptibility to human atherosclerosis through activation of autophagy and stimulation of cholesterol efflux. We demonstrate that perilipin‐2 modulates levels of the LXR ligand 27OH‐cholesterol and initiates a feed‐forward loop where LXR and autophagy reciprocally activate each other; the mechanism by which perilipin‐2 exerts its beneficial effects on subclinical atherosclerosis.

## Graphical abstract

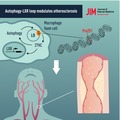

**Abbreviations**
3MA: 3‐methyladenine
27‐HC: 27‐hydroxycholesterol
CPIP: Carotid Plaque Imaging Project
IMT: carotid intima‐media thickness
LXR: liver‐X‐receptor
oxLDL: oxidized low‐density lipoprotein
PLIN2: perilipin‐2

## Introduction

Atherosclerotic cardiovascular disease (CVD) is the major cause of death worldwide. The natural history of atherosclerosis involves formation of macrophage foam cells and subsequent lesion formation in intermediate‐size and large arteries, characterized by lipid retention in the vessel wall, inflammation, cell death and fibrosis 1. Lipid droplets (LDs) are the major site of cholesterol storage in macrophage foam cells, and LD‐associated proteins are localized on the surface of LDs. Perilipin‐2 (PLIN2) is the most abundant LD‐associated protein in macrophage foam cells 2, 3 and has been implicated in atherosclerosis since it is highly expressed in macrophage foam cells 3 and *PLIN2* overexpression reduces cholesterol efflux from THP‐1 macrophages 4. Furthermore, *ApoE* ^−^ *^/^* ^−^ mice have ~3.5 times higher *PLIN2* expression, and genetic depletion of *PLIN2* in murine models of atherosclerosis results in reduced plaque burden 5. Thus, *PLIN2* is considered as a strong and promising target to treat atherosclerosis 3, 6. Recently, we have characterized a common protein variant in *PLIN2* (Pro251), which is associated with a less atherogenic plasma lipid profile 7, suggesting that this genetic variant may modulate macrophage foam cell formation and affect proneness to atherosclerosis.

The liver‐X‐receptor (LXR)‐dependent upregulation of cholesterol transporters and subsequent efflux of cholesterol to extracellular acceptors are an important process through which macrophages expel their excess cholesterol, which halts atherosclerosis 8, 9. In line with this, the ability of plasma to accept cholesterol expelled from macrophages, designated ‘cholesterol efflux capacity’, has been shown to be inversely associated with incidence of cardiovascular events 10. Autophagy is another significant contributor to cholesterol efflux from foam cells 11. Autophagy, which previously was primarily considered a cellular homoeostatic process 12, 13, has therefore been assigned a protective role in early atherosclerosis. Despite recent attention paid to autophagy in atherosclerosis and CVD 14, human data are still scarce and the relationship between LXR and autophagy in macrophage foam cells has never been explicitly examined.

In the present work, we sought to delineate the role of *PLIN2* in human atherosclerosis using the Ser251Pro variant in *PLIN2* as a genetic tool. We show that *PLIN2* modulates subclinical atherosclerosis, macrophage foam cell formation and cholesterol efflux by initiating a feed‐forward loop where LXR and autophagy reciprocally activate each other.

## Materials and methods

### Genetic association study

The IMPROVE study (acronym for ‘carotid intima‐media thickness (IMT) and IMT progression as predictors of vascular events in a high‐risk European population’ study) is a pan‐European, multicentre, longitudinal, cohort study designed to investigate whether cross‐sectional carotid IMT (C‐IMT) measurements and IMT progression are useful predictors of cardiovascular events in European individuals who are at high risk of CVD 15. The study comprises 3711 participants, aged 54–79 years, of whom 48% are men. Inclusion criteria were presentation of ≥3 classical cardiovascular risk factors and absence of previous events at enrolment. All participants have undergone state‐of‐the‐art high‐resolution carotid ultrasound examinations following an established protocol applied at all recruitment centres. In brief, the mean and maximum IMT measurements of the common carotid at the first centimetre proximal to the bifurcation, the common carotid (excluding the first centimetre proximal to the bifurcation), the carotid bifurcation and the internal carotid arteries were taken. IMT is defined as the thickness of the vessel wall, measured from the leading edge of the lumen‐intima interface to the leading edge of the media‐adventitia interface. Segment‐specific IMT measurements were used to generate composite IMT measurements; mean IMT, maximum IMT and the mean of the maximum IMT. C‐IMT variables were analysed both as baseline values and as changes over time (expressed in mm/year), calculated, within subject, by linear regression using 3 measurements taken at 0, 15 and 30 months. All study subjects gave their informed consent, and local ethics committees across all countries participating in the study approved the study. The entire cohort was genotyped for Ser251Pro single nucleotide polymorphism in PLIN2 using tailored TaqMan probes as previously described 7.

### Human carotid artery atherosclerotic plaques

Calculation of the % core area and CD68 staining using immunohistochemistry was carried out in 40 human carotid atherosclerotic plaques (20 from either variant of *PLIN2*, matched for age, sex and diabetes status and treatment) from the Carotid Plaque Imaging Project (CPIP). The CPIP biobank includes carotid plaques from patients undergoing carotid endarterectomies at the Skåne University Hospital, Malmö, Sweden. The indications for surgery were the presence of plaques associated with ipsilateral symptoms (transient ischaemic attack, stroke or amaurosis fugax) and stenosis higher than 70%, or plaques not associated with symptoms but with stenosis larger than 80% as previously described 16. Informed consent was obtained from each patient, and the local ethics review board approved the study.

### Isolation of lipoproteins

Lipoproteins used for foam cell formation, as well as for cholesterol efflux assays, were isolated through sequential ultracentrifugation from human plasma obtained from the Blood Central at Karolinska University Hospital. Briefly, plasma was ultracentrifuged for >22 h at 285,000 × ***g*** at 4°C. The upper‐most fraction containing chylomicrons was discarded, and the intermediate fraction containing low‐density lipoprotein (LDL) and high‐density lipoprotein (HDL) was collected. The density of the LDL/HDL fraction was adjusted to 1.063 g/ml with potassium bromide (Sigma‐Aldrich, Stockholm, Sweden) and ultracentrifuged as described. The upper fraction, now containing LDL, was collected and desalted using a PD‐10 column (GE Healthcare, Stockholm, Sweden). LDL was oxidized over night at 37°C using 20 μmol L^−1^ copper sulphate [CuSO4] (Merck), and the reaction was stopped using 1 mmol L^−1^ EDTA (Sigma‐Aldrich). The lower fraction containing HDL was desalted, sterile‐filtered and diluted to a concentration of 2 mg/mL and used as an acceptor in cholesterol efflux assays.

### Primary Monocytes and foam cell formation assay

Primary monocytes were isolated from whole blood of healthy individuals carrying either variant of *PLIN2* (9 noncarriers and 11 carriers were recruited). The recruit‐by‐genotype protocol has previously been described in detail 7. Briefly, whole blood was diluted 1:1 with phosphate‐buffered saline and layered onto a Ficoll‐Paque (GE Healthcare) gradient to separate monocytes/lymphocytes from red blood cells and neutrophils by centrifugation (400 × ***g***, 30 min). The intermediate fraction, containing monocytes and lymphocytes, was collected and residual red blood cells were lysed using Ammonium Chloride–Potassium (ACK) lysis buffer (Sigma‐Aldrich). A highly pure population of monocytes was obtained by magnetic depletion of nonmonocytic cells (Miltenyi Biotec, Lund, Sweden). When assessing the relationship between LXR and autophagy, monocytic cells were isolated using a hyper‐osmotic Percoll (GE Healthcare) solution and subsequent centrifugation (580 × ***g***, 15 min).

Monocytes at a density of 350,000 cells/mL were cultured on tissue culture plates using RPMI (+5% foetal bovine serum and 0.1% Penicillin‐Streptomycin) and differentiated with macrophage colony‐stimulating factor (M‐CSF) (100 ng/mL) treatment for 7 days, boosting the cells on day 3 with additional M‐CSF. When differentiated, macrophages were treated with an LXR agonist (GW3965, 10 μmol L^−1^), an LXR antagonist (GSK2033, 10 μmol L^−1^) or 25 μg/mL oxidized low‐density lipoprotein (oxLDL) for 24 h. Complete cell culture medium with dimethyl sulphoxide (DMSO) (in the case of GW3965 or GSK2033 treatment) or without supplementation with oxLDL served as control. When modulating autophagy, 2 h pre‐incubation with either a mammalian target of rapamycin (mTOR) inhibitor (rapamycin at 200 nmol L^−1^) or autophagy inhibitor (3‐methyladenine (3MA) at 5 mmol L^−1^) was adopted, upon which the cells were maintained in culture for 24 h. In order to monitor autophagy flux, 100 nmol L^−1^ bafilomycin A1 was added to each experimental condition as 2‐h pretreatment as indicated.

Human primary monocyte‐derived macrophages carrying either variant of *PLIN2* were challenged with oxLDL to generate foam cells; complete cell culture medium without oxLDL supplementation was used as control. Bafilomycin A1 was used to investigate whether autophagy affects foam cell formation. *PLIN2* expression was quantified using reverse transcription polymerase chain reaction (RT‐PCR) and Western blotting. Cholesterol ester accumulation was determined using a colorimetric assay and 27‐hydroxycholesterol (27‐HC) levels were assessed using mass spectrometry, as described.

Study subjects included in the recruit‐by‐genotype study gave their informed consent to their participation and the local ethics committee approved the study (approval nr. 02‐091).

### RT‐PCR and western blotting

RNA and protein were extracted from macrophage foam cells using RNeasy Mini Kits according to a modified manufacturer’s protocol, with the reagents provided (QIAGEN, Sollentuna, Sweden). After each centrifugation, the flow‐through was collected and pooled for biological replicates and protein was isolated from the flow‐through. RNA concentration was determined using NanoDrop 1000 (Thermo Scientific, Stockholm, Sweden). Reverse transcription was carried out using SuperScript III Reverse Transcriptase (Thermo Fisher Scientific) according to manufacturer’s instructions.

RT‐PCR was carried out using TaqMan gene expression assays (Thermo Fisher Scientific) and StepOne Plus Real‐Time PCR System (Thermo Fisher Scientific). Relative gene expression was determined using the Delta‐Delta‐CT (DDCT) method using RPLP0 (Hs999999902_m1) as reference gene.

Protein precipitated from flow‐through after storage at −20°C for >48 h and protein was pelleted by centrifugation at >15,000 ***g*** for 20 min at 4°C. Pellets were then dissolved in 8 mol L^−1^ urea before Western blotting. Protein extracts were loaded on 10% and 14% acrylamide sodium dodecyl sulphate–polyacrylamide gel electrophoresis (SDS‐PAGE) gels, for determination of PLIN2, p62 and LC3 protein expression. Upon migration, proteins were electrophoretically transferred to polyvinylidene difluoride membranes (BioRad, Solna, Sweden). Membranes were blocked in 5% milk in TBST (0.1% Tween‐20 in Tris‐buffered saline) to reduce nonspecific binding. Immunoblotting was carried out using anti‐PLIN2, p62 and LC3 antibodies diluted 1:2000 in 5% milk, or 1:1000 in 5% BSA in the case of p62 in TBST (Origene [Rockville, MD, USA], Santa‐Cruz and BioTechne Ltd., Oxon, England, UK, respectively). Horseradish peroxidase‐conjugated secondary antibodies (1:50,000, BioRad) were used to amplify the signal. Blots were developed using enhanced chemiluminescence reagent kit (GE Healthcare) and medical X‐ray films from AGFA. ImageJ was used for densitometry, and autophagy flux was defined as LC3‐II fold change between experimental conditions supplemented with bafilomycin A1 over experimental condition without bafilomycin A1 by using LC‐II density normalized to β‐actin.

### Gene expression profiling using microarrays

Gene expression profiling of monocyte‐derived macrophages and macrophage foam cells from individuals carrying either variant of *PLIN2* (*n* = 6) was carried out using the Clariom D microarray from Affymetrix. CEL files were preprocessed using Robust Multichip Averaging (RMA) normalization, including log2 transformation in Transcriptome Analysis Console (TAC) from Affymetrix. All downstream analyses were carried out in Bioconductor, R.

### Inflammatory characterization of macrophage foam cells

Cytokine production profiles were used to assess the inflammatory response of macrophage foam cells. Cell culture medium samples were taken at 0, 6 and 24 h upon oxLDL stimulation. Multiplexed ELISA was carried out on centrifuged medium supernatants using the Meso‐Scale Discovery (MSD) Proinflammatory Panel 1 (human) Kit, simultaneously quantifying 10 cytokines (interferon‐γ (INF‐γ), interleukin‐1β (IL‐1β), interleukin‐2 (IL‐2), interleukin‐4 (IL‐4), interleukin‐6 (IL‐6), interleukin‐8 (IL‐8), interleukin‐10 (IL‐10), interleukin‐12p70 (IL‐12p70), interleukin‐13 (IL‐13) and tumour necrosis factor (TNF‐α)). All diluents, calibrators and samples were prepared, and the assay was carried out according to the manufacturer’s instructions.

### Quantification of intracellular cholesterol and lipids

Lipids were extracted from the cell monolayers by addition of 2 mL hexane/isopropanol (3:2, v/v). Triglyceride (TG), total (TC) and unesterified cholesterol (UC) mass was measured by enzymatic assay using commercially available kits (Roche Diagnostics GmbH, Mannheim and Wako Chemicals, Richmond, VA). Cell cholesterol ester was calculated as the difference between TC and UC content. 27‐HC cholesterol levels were quantified by isotope dilution mass spectrometry as previously described 17. Total cell protein mass was measured using the RC DC™ Protein Assay (BioRad Laboratories Inc., USA) in cell monolayers digested with 1 mol L^−1^ NaOH.

### Cholesterol efflux assay

oxLDL was labelled with 2 μCi/mL ^3^H‐cholesterol (Perkin Elmer). Monocyte‐derived macrophages were loaded with ^3^H‐oxLDL in RPMI medium (+1 %FBS and 0.1% PEST) for 24 h. Cells were equilibrated for 2 h with 0.5 mL RPMI medium without or with the supplementation of 100 ng/mL bafilomycin A1 to inhibit autophagy. After equilibration, cells were washed 3 × 1 mL and 0.3 mL RPMI medium containing the indicated acceptor (20 μg/mL ApoA‐1 (Tebu‐bio) or 1 % total human serum) was added. As control, only RPMI medium without a cholesterol acceptor was used. Efflux was measured over 24 h, after which, the medium was collected from each well and the cells were lysed with 300 μL 0.1 mol L^−1^ NaOH. The collected medium and cell lysates were centrifuged at 16,000 × ***g*** for 10 min, and cholesterol efflux was determined by scintillation counting.

Protein mass of the cell lysates was determined using the micro Bradford assay in accordance with the manufacturer’s instructions (BioRad). Cholesterol efflux data are adjusted to total protein content in cell lysates (% Efflux*/*μg Protein).

### Human embryonic kidney cells

Human embryonic kidney 293 (HEK293) cells were transfected with human *PLIN2* constructs containing either the major allele sequence (Ser251) or the minor allele sequence (Pro251) using lipofectamine 2000 (Thermo Fisher Scientific), as described in detail 7. Stable clones were selected by culturing transfected cells with 600 μg/mL Geneticin (Thermo Fisher Scientific) and then maintained in 50 μg/mL Geneticin. Clones were selected by assessing PLIN2 protein expression (Western blot), as previously described. All treatments on HEK293 cells followed stabilization of PLIN2 protein using a lipid‐loading medium containing 400 µmol L^−1^ oleic acid.

Co‐transfection of 100 ng of a 3xLXR responsive element (LXRE) luciferase reporter and renilla luciferase was carried out using Lipofectamine 3000 transfection reagent (Thermo Fisher Scientific) as previously described 18. Data are presented as LXRE luciferase activity normalized to renilla luciferase.

### Statistics

Genetic data were analysed using SPSS and Plink software v.1.07 19 to generate linear regression models, assessing the effect of rs35568725 on C‐IMT phenotypes. Covariates included age, sex, plasma lipid levels and multi‐dimensional scaling (MDS) dimensions 1‐3 to adjust for population substructures over the pan‐European population.

Experimental data were plotted and analysed using nonparametric tests in GraphPad Prism 7; all data are either presented as mean ± standard error (SEM) or median and inter‐quartile range (IQR) as indicated. *P*‐values <0.05 were considered significant and multiple‐testing adjustments were applied where appropriate.

## Results

### PLIN2 functionality has significant consequences on subclinical atherosclerosis and plaque formation

Since we have previously shown that the Pro251 variant of *PLIN2* is associated with a more profitable plasma lipid profile and *PLIN2* initiates murine atherosclerosis, we investigated whether *PLIN2* modulates development of human atherosclerosis. A high‐risk pan‐European population, which had undergone high‐resolution ultrasonographic investigation of IMT in the carotid arteries, was genotyped for the *PLIN2* Ser251Pro polymorphism. The Pro251 allele was associated with decreased carotid IMT and slower IMT progression over 30 months compared to the major Ser251 variant. Specifically, the Pro251 allele was moderately associated with decreased carotid IMT measured as Mean–Max IMT at baseline (β = −0.009, *P* = 0.04) and internal carotid (β = −0.012, *P* = 0.003) as well as mean carotid artery IMT (β = −0.003, *P* = 0.05) over 30 months of follow‐up compared to the major Ser251 allele, Table 1. These associations were independent of plasma lipid levels since correction for plasma lipid levels did not affect the results.

**Table 1:** PLIN2 modulates subclinical atherosclerosis

CHR	SNP	Major allele	Minor allele	C‐IMT‐phenotype	MAF	BETA	SE	P
C‐IMT at Baseline								
9	rs35568725	Ser251	Pro251	Mean–Max IMT	0.05	−0.009	0.005	0.04
9	rs35568725	Ser251	Pro251	Mean IMT	0.05	−0.0103	0.006	0.07
9	rs35568725	Ser251	Pro251	Mean ICA IMT	0.05	−0.0156	0.018	0.39
C‐IMT change over time								
9	rs35568725	Ser251	Pro251	Mean–Max IMT	0.05	−0.0021	0.003	0.47
9	rs35568725	Ser251	Pro251	Mean IMT	0.05	−0.003	0.002	0.05
9	rs35568725	Ser251	Pro251	Mean ICA IMT	0.05	−0.012	0.004	0.003

Association of rs35568725 with C‐IMT at baseline and change over time up until 30 months of follow‐up in the IMPROVE study. Chromosome (CHR); single nucleotide polymorphism (SNP); minor allele frequency (MAF); beta value for regression model (BETA); standard error (SE); *P*‐value for association (P).

**Figure 1:**
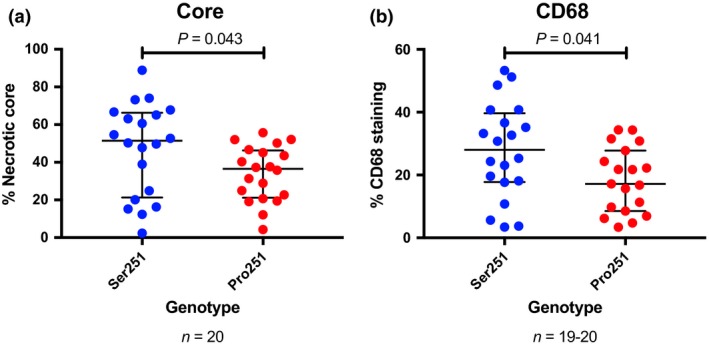
PLIN2 functionality has significant consequences on carotid plaque growth and macrophage infiltration. (a) The % core area and (b) CD68 staining using immunohistochemistry of 40 human carotid atherosclerotic plaques from the Carotid Plaque Imaging Project carrying either variant of PLIN2, matched for age, sex and diabetes status. Data are presented as median and IQR.

Since larger necrotic cores and increased infiltration of CD68‐positive cells are indicative of a vulnerable plaque, core and CD68 area of carotid atherosclerotic plaques originating from patients undergoing carotid endarterectomy were analysed with respect to *PLIN2* genotype. Carriers of the Pro251 variant presented with an approximately 25% reduction plaque core area and plaque CD68 content, indicating a slower growing plaque compared with noncarriers (*P* = 0.043 and *P* = 0.041, respectively), Fig. 1. These data, in concert with the findings on carotid IMT, suggest that the Pro251 allele in *PLIN2* possesses properties protective of atherosclerosis development.

### Cholesteryl ester accumulation and 27OH‐cholesterol (27‐HC) are modulated by PLIN2 functionality in oxLDL‐treated monocyte‐derived macrophages

We utilized the functional Ser251Pro variant in *PLIN2* as a genetic tool to investigate the mechanism behind the protective effects observed on C‐IMT.

**Figure 2:**
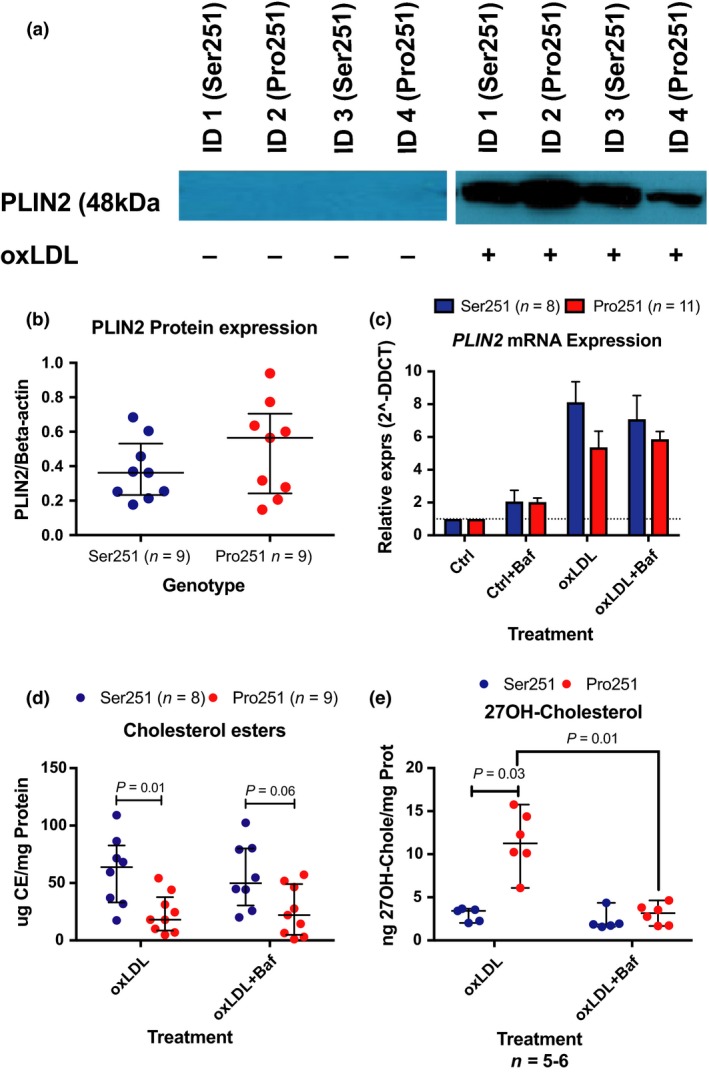
PLIN2 modulates the availability of 27‐HC and thereby also LXR activity. (a–c) PLIN2 expression by variant in PLIN2 as shown by Western blotting and qPCR. (d–e) Cholesterol ester and 27‐HC accumulation upon oxLDL challenge. Data are presented as median and IQR (b, d, e), and mean ± SEM (c). See also Fig. S1.

Individuals carrying either variant of *PLIN2* were recruited, and primary monocyte‐derived macrophages were prepared from whole blood and were treated with or without oxLDL. Independently of genotype, PLIN2 protein was undetectable in untreated primary monocyte‐derived macrophages, but was readily stabilized upon oxLDL challenge, Fig. 2(a). Human primary monocyte‐derived macrophages carrying the different variants of *PLIN2* had similar levels of mRNA and protein expression of *PLIN2*, Fig. 2(a–c). Although both variants show similar PLIN2 protein levels, human primary monocyte‐derived macrophages carrying the Pro251 variant of *PLIN2* showed a 50% reduction of cholesteryl ester accumulation upon oxLDL challenge compared to macrophages homozygous for the major variant (*P*= .01), Fig. 2(d). Contrasting, macrophages carrying Pro251 displayed more than a 2‐fold increase in 27‐HC levels (*P* = 0.03), Fig. 2(e).

Since *PLIN2* is located in the crossroads of lipid metabolism and autophagy, we investigated the influence of autophagy in this setting. Deregulation of autophagy by adding bafilomycin A1 did not alter *PLIN2* expression, nor cholesteryl ester accumulation, but normalized 27‐HC levels in primary monocyte‐derived macrophages carrying the Pro251 variant to the levels of Ser251, Fig. 2(b–e). Thus, *PLIN2* functionality modulates the availability of 27‐HC.

### PLIN2 influences LXR activity and cholesterol efflux by autophagy in monocyte‐derived macrophages

**Figure 3:**
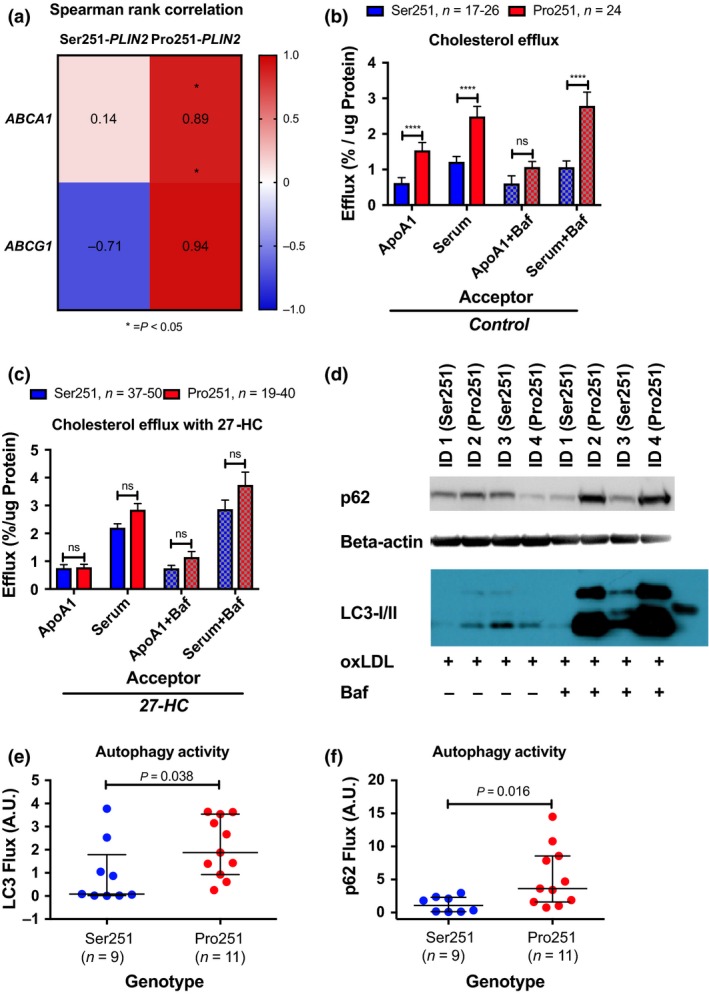
PLIN2 comprises a hub in cholesterol metabolism by connecting LXR activity and autophagy. (a) LXR target co‐expression by PLIN2 variant. (b) Cholesterol efflux without 27‐HC supplementation to the respective acceptor by protein variant of PLIN2. (c) Cholesterol efflux with 27‐HC supplementation to the respective acceptor by protein variant of PLIN2. (d) Autophagy activity, displayed as LC3 and p62 flux, by PLIN2 variant using Western blotting. (e, f) Densitometry of bands obtained from Western blotting. Autophagy blockade using bafilomycin A1 was applied as indicated. Data are presented as mean ± SEM (b), and median and IQR (d, e). **P < 0.01, ***P < 0.001, ****P < 0.0001. See also Fig. S2.

In the light of the increased levels of 27‐HC in oxLDL‐treated monocyte‐derived macrophages carrying the Pro251 allele compared to Ser251 allele, and the fact that 27‐HC is an endogenous ligand for LXR, we hypothesized that LXR activity was influenced by the polymorphism. Significant co‐expression patterns were found between Pro251‐*PLIN2* mRNA and mRNA of *ABCA1* and *ABCG1*. In contrast, Ser251‐*PLIN2* mRNA was not co‐expressed with these cholesterol transporters, Fig. 3(a).

Because co‐expression patterns do not necessarily reflect function, cholesterol efflux to apoA1 and total serum from monocyte‐derived macrophage foam cells was studied. Cells carrying the Pro251 variant had approximately 2‐fold higher cholesterol efflux to apoA1 and total serum, compared to monocyte‐derived macrophages carrying the major variant (*P* < 0.001, Fig. 3(b). Observed effects on cholesterol efflux were partly dependent on intact autophagy since deregulation of autophagy by bafilomycin A1 supplementation ablates the beneficial effects of the Pro251 variant on cholesterol efflux to apoA1, Fig. 3(b). Ablation of the beneficial effects of Pro251 was accomplished by supplementing this system with 2.5 μmol L^−1^ 27‐HC, further the effect of autophagy was diminished, Fig. 3(c).

In line with data herein, autophagy flux was augmented by ~2‐fold in oxLDL‐treated human primary monocyte‐derived macrophages carrying Pro251 compared to noncarrying cells, as measured by LC3 lipidation and p62 accumulation upon bafilomycin A1 supplementation (*P* = 0.038 and *P* = 0.016, respectively), Fig. 3(d–f)*, *see also Fig. S2A.

Additionally, the immunophenotype of monocyte‐derived macrophages was assessed using a co‐expression approach as well as 10‐plex electrochemiluminescense assay for cytokine production. Moderate mRNA co‐expression patterns were found between the M2 markers *IL10* and *ARG1* and Pro251‐*PLIN2,* whereas 251Ser‐*PLIN2* was co‐expressed with pro‐inflammatory and phagocytic markers *IL6* and *CD68*. Interestingly, IL‐10, generally considered an M2 marker and an anti‐inflammatory cytokine, was increased in cell culture media from oxLDL‐treated monocyte‐derived macrophages carrying Pro251 compared to Ser251. Similarly, to cholesterol efflux, this effect on IL‐10 was, in part, dependent on intact autophagy, Fig. S2B‐D.

Taken together, data suggest that *PLIN2* comprises a hub in cholesterol metabolism by connecting LXR activity and autophagy.

### PLIN2 modulates LXR activation with significant repercussions on autophagy activity

Collectively, the Pro251 variant in *PLIN2* was associated with higher levels of 27‐HC, with a modulation of LXR target gene expression and with increased cholesterol efflux, which were in part dependent on intact autophagy. Hence, autophagy may be a means by which LXR activity is fine‐tuned, through 27‐HC. We thus sought to investigate whether the two variants of *PLIN2* presented with different levels of LXR activation, and whether autophagy regulates LXR activity.

**Figure 4:**
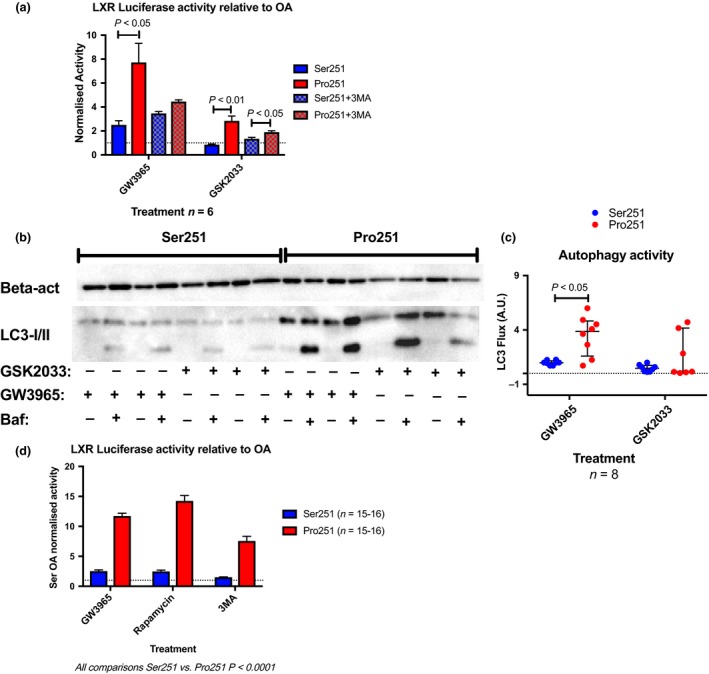
An in vitro system of HEK293 cells confirms that the functional PLIN2 protein variant modulates LXR activity. LXR was stimulated or inhibited using GW3965 and GSK2033, and autophagy was either stimulated or inhibited as indicated. (a) LXR luciferase activity in HEK293 cells carrying either variant of PLIN2. (b, c) Autophagy activity, displayed as LC3 flux, in response to LXR activation. (d) LXR Luciferase activity in response to rapamycin and 3‐methyladenine (3MA) treatment. Data are presented as mean ± SEM (a, d), and median and IQR (c). See also Fig. S2.

Stably transfected HEK293 cells carrying either variant of *PLIN2* were transfected with a luciferase reporter construct carrying an LXR responsive element. Luciferase activity was investigated in response to LXR activation and inhibition, respectively. Firefly luciferase activity was readily induced by treatment with GW3695. Analogous to previous data herein, the response was significantly higher in cells carrying Pro251 (7‐fold for Pro251 vs. 2‐fold for Ser251, *P* < 0.05), Fig. 4(a) (solid bars). Interestingly, early autophagy blockade using 3MA resulted in a blunted response to the LXR agonist to 60% of the activity of GW3695 treatment alone in cells carrying Pro251 allele, Fig. 4(a)* (checked bars)*. In addition, LXR stimulation resulted in an ~7‐fold upregulation of *CYP27A1* mRNA in HEK cells carrying the Pro251 variant (p = 0.0007), Fig. S3. Notably, HEK cells carrying the Pro‐variant of *PLIN2* presented with roughly 3‐fold increase in autophagy flux when LXR is activated (*P* = 0.01, Ser251 vs. Pro251), an effect ablated by treating the cells with an LXR antagonist (*P* = 0.96, Ser251 vs. Pro251), Fig. 4(b,c).

To corroborate the notion that autophagy modulates LXR activity, the influence of autophagy stimulation on the activity of the LXR responsive element was assessed. Irrespectively of genotype, treatment with the mTOR inhibitor rapamycin resulted in an at least 3‐fold increase in luciferase activity compared to control condition (*P* = 0.0035 and 0.0022 for Ser251 and Pro251, respectively), Fig. 4(d). Differences in absolute luciferase activity prevailed between the *PLIN2* protein variants.

### Crosstalk between LXR and autophagy

**Figure 5:**
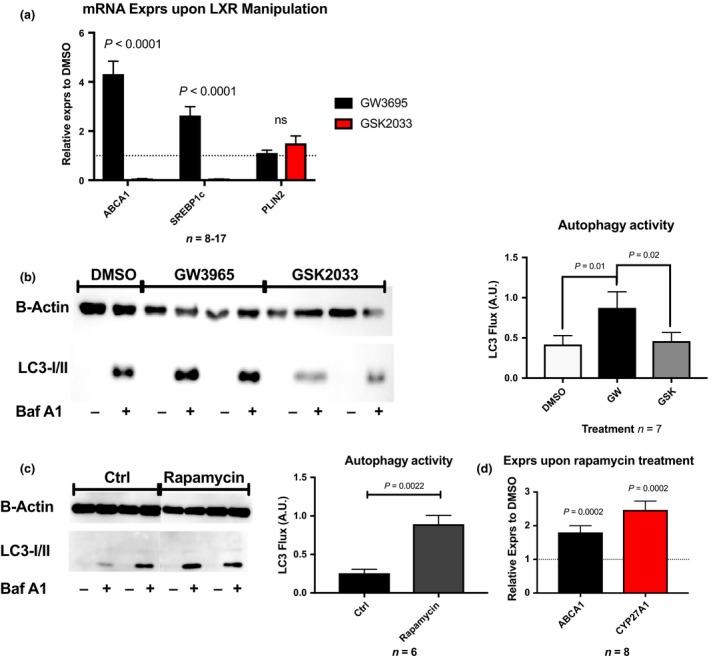
LXR and autophagy regulates their reciprocal activation in human primary monocyte‐derived macrophages. (a) mRNA expression of LXR targets SREBP1c and ABCA1, and PLIN2 upon manipulation of LXR activity using GW3965 and GSK2033. (b) Autophagy activity, displayed as LC3 flux, upon manipulation of LXR activity using GW3965 and GSK2033. (c, d) Gene expression and autophagy activity, displayed as LC3 flux, in monocyte‐derived macrophages induced by rapamycin stimulation. Data are presented as mean ± SEM.

Human primary monocyte‐derived macrophages were treated with the LXR agonist or LXR antagonist in order to determine the reciprocal influences between LXR and autophagy in a relevant cell type. As expected, GW3965 stimulation induced mRNA expression of both *SREBP1c* and *ABCA1* 3.5‐4‐fold, whereas LXR inhibition abolished their expression. *PLIN2* mRNA expression remained unchanged, Fig. 5(a). Interestingly, LXR stimulation resulted in a 2‐fold increase in autophagy activity (*P* = 0.01), measured as LC3 lipidation after bafilomycin A1 supplementation compared to DMSO control. LXR inhibition using GSK2033 normalized autophagy activity to levels of the control (*P* = 0.02), Fig. 5(b). Thus, these results suggest that LXR activity controls the level of activity of autophagy in human primary monocyte‐derived macrophages.

Monocyte‐derived macrophages were treated with the mTOR inhibitor rapamycin to stimulate autophagy activity to assess whether autophagy *per se* controls the activity of LXR. LC3 lipidation was induced 3‐fold by rapamycin treatment compared to DMSO‐treated controls (*P* = 0.002), Fig. 5(c). The induction of autophagy also resulted in an almost 2‐fold upregulation of LXR target genes *ABCA1* and *CYP27A1* mRNA (*P* = 0.0002, Fig. 5(d).

Collectively, data indicate that LXR and autophagy are responsible for their reciprocal activation.

### Autophagy generates the endogenous LXR ligand 27‐HC, which in turn stimulates autophagy

**Figure 6:**
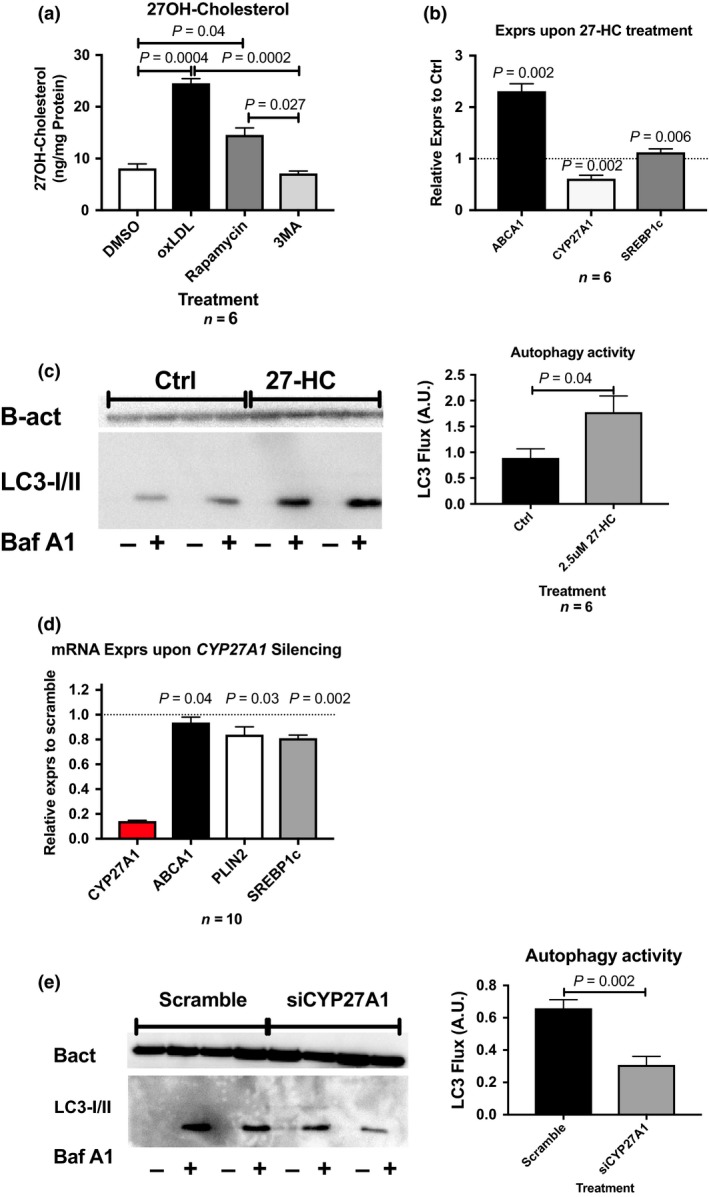
27‐HC serves as a pivotal link in the crosstalk between LXR and autophagy in human primary monocyte‐derived macrophages. (a) 27‐HC measurements in response to oxLDL, rapamycin or 3‐methyladenine. (b) mRNA expression LXR targets SREBP1c, ABCA1 and CYP27A1 upon 27‐HC treatment. (c) Autophagy activity, displayed as LC3 flux, upon 27‐HC treatment. (d) mRNA expression LXR targets SREBP1c, ABCA1 and CYP27A1, upon CYP27A1 silencing. (e) Autophagy activity, displayed as LC3 flux, upon CYP27A1 silencing. Data are presented as mean ± SEM.

Since rapamycin treatment upregulated *CYP27A1* expression and *CYP27A1* encodes the enzyme generating 27‐HC, we also investigated whether autophagy activity regulates 27‐HC content. Monocyte‐derived macrophages were treated with oxLDL, rapamycin or 3MA, to stimulate foam cell formation, activate or inhibit autophagy, respectively. Treatment with oxLDL increased 27‐HC content more than 2‐fold (*P* = 0.0004), as anticipated. Autophagy stimulation using rapamycin led to a 1.5‐fold increase of 27‐HC levels compared to DMSO‐treated control cells (*P* = 0.04). Autophagy inhibition using 3MA normalized 27‐HC levels to that of DMSO treatment (*P* = 0.027 vs. rapamycin), Fig. 6(a).

Treatment of monocyte‐derived macrophages with 27‐HC resulted in a 2‐fold increase in expression of the LXR target gene *ABCA1* (*P* = 0.002) and a 20% increase in *SREBP1c* mRNA (*P* = 0.0065), which was paralleled by a 50% downregulation of *CYP27A1* gene expression (*P* = 0.002), Fig. 6(b). Interestingly, the upregulation of LXR target genes by 27‐HC treatment was accompanied by an ~1.5‐fold increase in autophagy activity, which was comparable to that observed in monocyte‐derived macrophages stimulated with the LXR agonist GW3965 (*P* = 0.04), Fig. 6(c). Ablation of *CYP27A1* mRNA expression moderately blunted the expression of *ABCA1*, *SREBP1c* and *PLIN2 *Fig. 6(d), which was accompanied by an almost 70% reduction of autophagy activity (*P* = 0.002, Fig. 6(e).

Consequently, *CYP27A1* and 27‐HC constitute an important link in the crosstalk between LXR and autophagy, which is regulated by *PLIN2*.

## Discussion

For the first time, we show that the Pro251 variant in *PLIN2* is associated with decreased subclinical atherosclerosis as well as smaller necrotic core sizes and decreased macrophage infiltration in advanced atherosclerotic plaques. Our data propose that *PLIN2* modulates a feed‐forward loop where LXR and autophagy reciprocally activate each other, which ultimately has repercussions on foam cell formation, cholesterol efflux and development of subclinical atherosclerosis progression.

The formation of macrophage foam cells, as a result of increased cholesterol accumulation, constitutes one of the initial steps in the formation and growth of atheromatous lesions. Since *PLIN2* is central for macrophage foam cell formation and we have previously shown that a *PLIN2* variant influences plasma lipid profiles 7, we hypothesized that the Pro251 variant in *PLIN2* would affect atherosclerosis development. Carotid IMT is an established risk factor for future cardiovascular events 15, and by adopting a molecular genetic approach, we here show that *PLIN2* is influencing subclinical atherosclerosis as measured by C‐IMT. Histologically, *PLIN2* has an impact on the core area and macrophage infiltration in human carotid plaques and collectively, past and present data support the notion of *PLIN2* modulating cardiovascular risk 4, 7, 20.

Cholesterol efflux from macrophage foam cells counters the expansion of atherosclerotic plaques, and this has been suggested as a therapeutic leverage in the treatment of atherosclerosis 21. *ABCA1* plays a key role in cholesterol efflux, transferring phospholipids and cholesterol from the cellular membranes to apolipoprotein A‐I and HDL particles 22, 23. The importance of this process in human has been shown by the observation that an effective *ABCA1*‐mediated cholesterol efflux protects from cardiovascular events 10. LXR is one of the major transcriptional regulators of *ABCA1* expression 24, and LXR deficiency leads to macrophage foam cell formation that is paralleled by increased LDL and reduced HDL cholesterol 25. Autophagy is another important contributor to cholesterol efflux from macrophage foam cells. The process mobilizes LDs to lysosomes in which lysosomal acid lipases generate free cholesterol available for efflux from the cell 11. Free cholesterol can be oxidized into oxysterols; molecules that strongly activate LXR 26. When comparing monocyte‐derived macrophages from donors bearing the *PLIN2* Pro251‐variant, to cells bearing the Ser251‐variant, they display not only increased cholesterol efflux leading to lower cholesteryl ester levels, but also increased levels of 27‐HC, an effect dependent on intact autophagy. Since the substitution at residue 251 does not occur in the promoter region of the *PLIN2* gene, and we have shown that human primary monocyte‐derived macrophages carrying the different variants of *PLIN2* have similar expression of *PLIN2*, this suggests conserved transcriptional activation by cholesterol/oxysterol and LXRs. The increase in hydrolysis of cholesteryl esters brought about by Pro251 leads to an increase of free cholesterol in the cell, which in turn determines synthesis of 27‐HC, as free cholesterol is the substrate for 27‐HC synthesis. Deregulation of autophagy by supplementing bafilomycin A1 did not alter *PLIN2* expression, nor cholesteryl ester accumulation, but decreased 27‐HC levels in primary monocyte‐derived macrophages carrying the Pro251 variant to the levels of Ser251. Hence, *PLIN2* seems to be able to modulate only the synthesis of 27‐HC via autophagy, whereas the effects on the cellular levels of cholesteryl esters seem to be independent of autophagy.

Significant co‐expression of the Pro251‐*PLIN2* mRNA and the expression of LXR target genes – *ABCA1* and *ABCG1 –* show that the ligand generated by the increased autophagy associated with the *PLIN2* Pro251 variant activates LXR. Thus, by studying cells from individuals bearing different *PLIN2* genetic variants, we clearly demonstrate that LXR and autophagy crosstalk. Nevertheless, autophagy cannot entirely explain the differences in LXR activation and cholesterol efflux observed between the two *PLIN2* genetic variants because its blockade only blunts the effects, not ablates them. Our observations made in human primary monocyte‐derived macrophage foam cells were also reproduced in another *in vitro* system, using stably transfected HEK293 cells carrying either genetic variant of *PLIN2*. By monitoring *CYP27A1* mRNA expression, LXRE luciferase activity and autophagy, it was also clear that the effects on autophagy observed in cells carrying the *PLIN2* Pro251 variant were LXR‐dependent as inhibition of LXR ablated differences between the variants of *PLIN2* with regards to autophagy flux. The use of rapamycin in stably transfected HEK293 cells determined an increase in LXRE luciferase activity, which was dependent on intact autophagy. The interconnection between LXR and autophagy was also evident in human primary monocyte‐derived macrophages: (i) by using rapamycin, a molecule that does not directly activate LXR, which increased the expression of the LXR target gene *ABCA1*; (ii) by directly stimulating LXR with GW3965, which increased autophagy and, obviously, also the mRNA expression of *SREBP1c* and *ABCA1*; (iii) by perturbation of *CYP27A1*, the gene encoding the enzyme generating the LXR ligand 27‐HC, which is alone sufficient to reduce autophagy activity. The studies with rapamycin also corroborated the concept that synthesis of 27‐HC is in part dependent of autophagy, since its stimulation using this mTOR inhibitor increased the expression of *CYP27A1*, leading in turn to increased levels of the endogenous LXR ligand 27‐HC. Therefore, it seems that autophagy and LXR reciprocally regulate each other by a feed‐forward loop through 27‐HC.

Several lines of evidence suggest that both LXR and autophagy activation are protective against CVD, not only by modulating cholesterol metabolism but also by modulation of macrophage immunophenotypes, promotion of effective efferocytosis and resolving inflammation 11, 12, 13, 27, 28, 29. The observations that the *PLIN2* Pro251 variant is associated with increased IL‐10 production and that its expression correlates with M2 macrophage mRNA markers further extend the notion of crosstalk between autophagy and LXR in the modulation of the inflammatory responses.

In summary, we demonstrate that *PLIN2* modulates subclinical atherosclerosis by reducing lipid retention in the vascular wall and macrophage infiltration. The mechanism by which these beneficial effects are produced seems to be dependent on activation of a feed‐forward loop between LXR and autophagy. Our hypothesis is supported by mechanistic studies in monocyte‐derived macrophage foam cells carrying different *PLIN2* genetic variants, and from an *in vitro* model of stably transfected cells carrying either variant of *PLIN2*. The crosstalk between LXR and autophagy is mediated by 27‐HC and has a striking impact on foam cell formation by promoting cholesterol efflux, decreasing cholesteryl ester accumulation and improving macrophage immunophenotypes. Ultimately, the intricate molecular relationship between LXR and autophagy has repercussions on atherosclerosis progression and deserves future attention.

## Conflict of interest

The authors declare no competing interests pertaining the present study. The manuscript has been handled by an external editor, Professor of Medicine Sam Schulman, Mac Master University, Hamilton, Ontario, Canada.

## Supporting information


**Figure S1** **.** Characterisation of foam cell formation assay.
**Figure S2** **.** Autophagy modulates monocyte‐derived macrophage foam cell immunophenotypes.
**Figure S3** **.** CYP27A1 is increased in HEK293 cells carrying the Pro251 variant in *PLIN2*.

## Appendix 1

### The IMPROVE Study Group

- Dipartimento di Scienze Farmacologiche e Biomolecolari, Universita di Milano, Milan, Italy: C. R. Sirtori, S. Castelnuovo.
- Centro Cardiologico Monzino, IRCCS, Milan Italy: M. Amato, B. Frigerio, A. Ravani, D. Sansaro, C. Tedesco, F. Bovis, A. Discacciati.
- Atherosclerosis Research Unit, Departments of Medicine and Cardiology, Karolinska University Hospital Solna, and Division of Cardiovascular Epidemiology, Institute of Environmental Medicine, Karolinska Institutet, Stockholm, Sweden: M. Ahl, G. Blomgren, M. J. Eriksson, P. Fahlstadius, M. Heinonen, L. Nilson.
- University College of London, Department ofMedicine, Rayne Institute, London, United Kingdom: J. Cooper, J. Acharya.
- Foundation for Research in Health Exercise and Nutrition, Kuopio Research Institute of Exercise Medicine, Kuopio, Finland: K. Huttunen, E. Rauramaa, H. Pekkarinen, I. M. Penttila, J. Torronen.
- Department of Medicine, University Medical Center Groningen, Groningen and Isala Clinics Zwolle, Department of Medicine, the Netherlands: A. I. van Gessel, A. M. van Roon, G. C. Teune, W. D. Kuipers, M. Bruin, A. Nicolai, P. Haarsma‐Jorritsma, D. J. Mulder, H. J. G. Bilo, G. H. Smeets.
- Assistance Publique—Hopitaux de Paris; Service Endocrinologie‐ Metabolisme, Groupe Hopitalier Piti.‐Salpetriere, Unites de Prevention Cardiovasculaire, Paris, France: J. L. Beaudeux, J. F. Kahn, V. Carreau, A. Kontush.
- Institute of Public Health and Clinical Nutrition, University of Eastern Finland, Kuopio Campus: J. Karppi, T. Nurmi, K. Nyyssonen, R. Salonen, T. P. Tuomainen, J. Tuomainen, J. Kauhanen.
- Internal Medicine, Angiology and Arteriosclerosis Diseases, Department of Clinical and Experimental Medicine, University of Perugia, Perugia, Italy: G. Vaudo, A. Alaeddin, D. Siepi, G. Lupattelli, G. Schillaci.
