# Supplementary material for: Subclinical atherosclerosis and its progression are modulated by PLIN2 through a feed‐forward loop between LXR and autophagy
Source: J Intern Med. 2019 Jul 29;286(6):660–75. doi: 10.1111/joim.12951 (PMC6899829; doi:10.1111/joim.12951)

**Characterisation of foam cell formation assay.**

Supplementary Figure 1. A-D) *PLIN2* and cholesterol transporters' mRNA expression upon oxLDL challenge. E-F) mRNA expression of genes involved in lipid synthesis upon oxLDL challenge. Data are presented as mean±SEM.

**Autophagy modulates monocyte-derived macrophage foam cell immunophenotypes**

Supplementary Figure 2. A) Autophagy activity, displayed as LC3 flux, in untreated monocyte-derived macrophages carrying either protein variant of *PLIN2*. B) Co-expression patters between *PLIN2* and inflammatory and M2 macrophage markers by *PLIN2* protein variant. C-D) Immunophenotyping of macrophage foam cells carrying either variant of *PLIN2*. Autophagy was inhibited as indicated. Data are presented as median and IQR (A), and mean±SEM (C-D).

**CYP27A1 is increased in HEK293 cells carrying the Pro251 variant in *PLIN2***

Supplementary Figure 3. A) *CYP27A1* mRNA expression upon GW3965 treatment. Data are presented as mean±SEM.

# Supplementary Figure 1

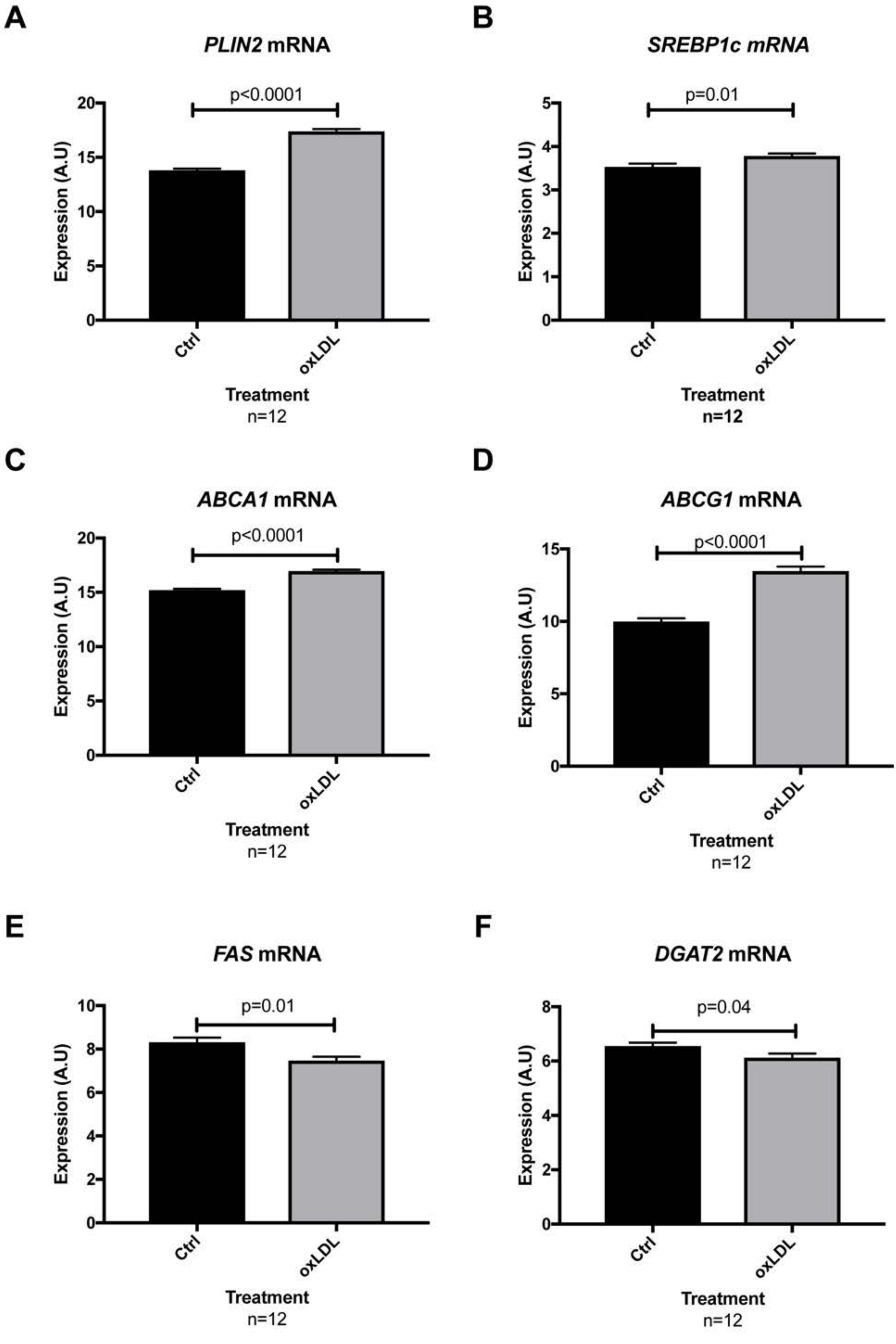

# Supplementary Figure 2

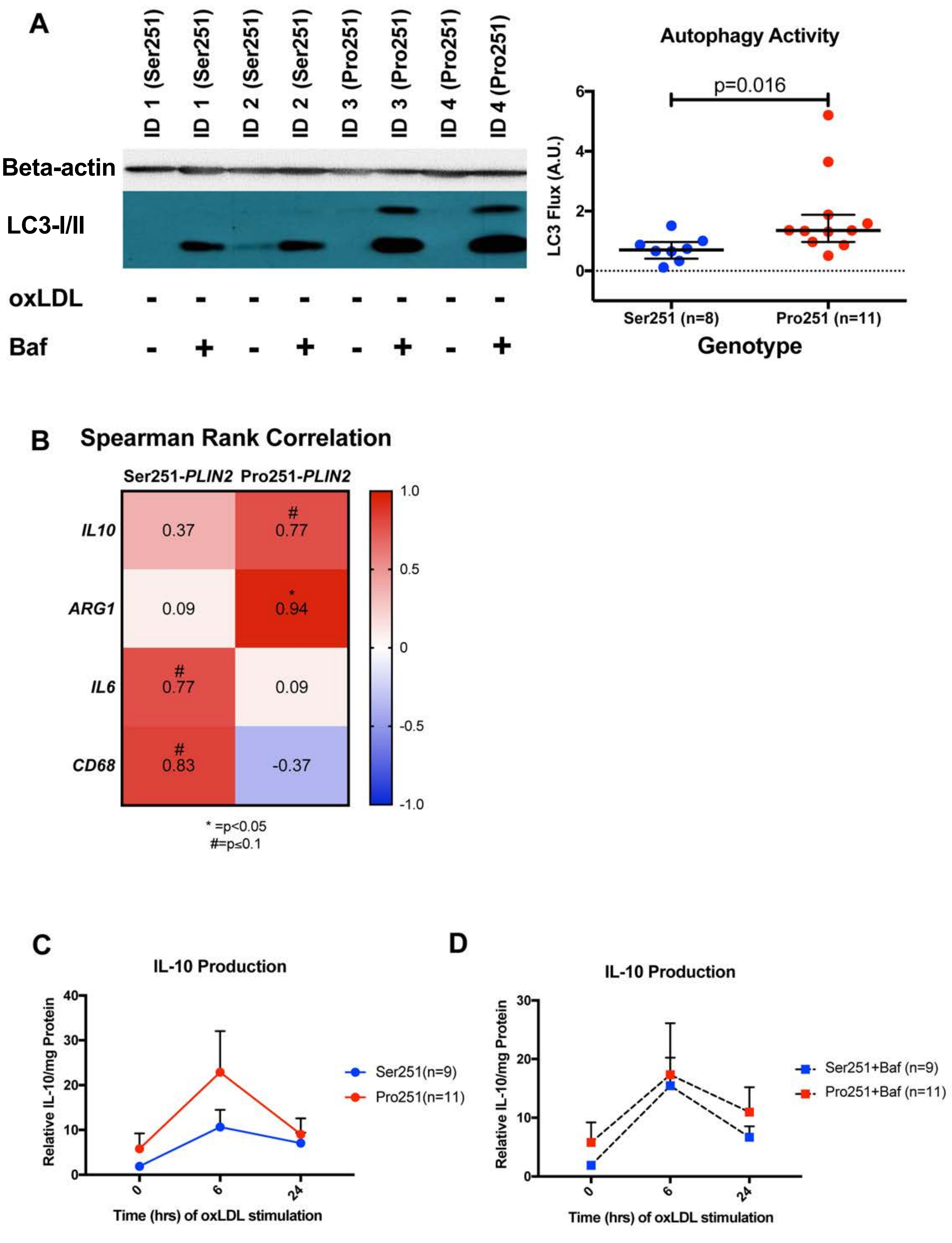

# Supplementary Figure 3

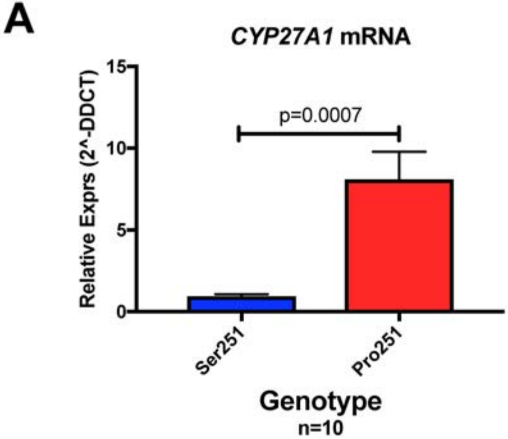

Supplement: Supplementary file 1 — Figure S1 . Characterisation of foam cell formation assay. Figure S2 . Autophagy modulates monocyte‐derived macrophage foam cell immunophenotypes. Figure S3 . CYP27A1 is increased in HEK293 cells carrying the Pro251 variant in PLIN2. [file JOIM-286-660-s001.pdf]
